# Cerebellar encephalitis associated with anti-mGluR1 antibodies: a case report and comprehensive literature review

**DOI:** 10.3389/fneur.2024.1333658

**Published:** 2024-02-12

**Authors:** Xue Chen, Yanan Chen, Lu Di, Na Liu, Ting Liu, Yun Cai, Weiying Di

**Affiliations:** ^1^Department of Neurology, Affiliated Hospital of Hebei University, Baoding, China; ^2^Hospital of Stomatology Hebei Medical University, Shijiazhuang, China

**Keywords:** cerebellar encephalitis, anti-mGluR1 antibodies, case report, EBV, literature review

## Abstract

Anti-metabotropic glutamate receptor 1 encephalitis is an uncommon autoimmune condition characterized by a subacute onset of cerebellar syndrome. Frequently, it also manifests as sleep disorders and cognitive or behavioral changes. While immunotherapy is the primary treatment approach, the disease remains poorly understood. Herein, we present a case of anti-metabotropic glutamate receptor 1 encephalitis, highlighting its primary cerebellar syndrome manifestation. The first magnetic resonance imaging scan showed no obvious abnormality. Lumbar puncture showed increased cerebrospinal fluid pressure, increased white blood cell count and protein level. The next-generation sequencing of cerebrospinal fluid showed Epstein–Barr virus infection, and the patient was diagnosed with viral cerebellar encephalitis. However, antiviral therapy was ineffective. Finally, anti-metabotropic glutamate receptor 1 was measured at 1:1,000, and the patient was definitely diagnosed with anti-metabotropic glutamate receptor 1 encephalitis. Therefore, clinicians should pay attention to such diseases to avoid misdiagnosis.

## Introduction

Anti-metabotropic glutamate receptor 1 (mGluR1) encephalitis is a seldom-encountered autoimmune disorder impacting both the central and the peripheral nervous system. It primarily instigates an acute or subacute cerebellar syndrome with varying severity. mGluRs are G-protein-coupled receptors situated both pre-and post-synaptically across the central and peripheral nervous systems, predominantly expressed in Purkinje cells. Their roles span cerebellar development, synaptic transmission modulation, synaptic plasticity, pain perception, memory, learning, and anxiety management (1). mGluR1 activation fosters long-term depression in parallel fiber-Purkinje cell synapses, a pivotal process for cerebellar motor learning (2). In this report, we present a case of cerebellar encephalitis associated with anti-mGluR1.

## Case presentation

**Figure 1:**
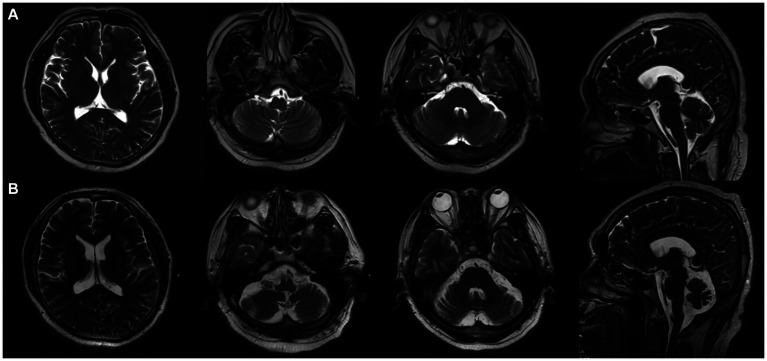
On 25 March 2020 **(A)**, cranial MRI showed no abnormality, and on 20 April 2023 **(B)**, cranial MRI showed cerebellar atrophy.

A 50-year old male laborer with a 15-year history of hypertension was admitted to our facility on 25 March 2020, presenting with symptoms of fever, dizziness, slurred speech, and unsteady gait persisting for 20 days. About 20 days before admission, he had shown a peak temperature of 37.5°C accompanied by the same neurological symptoms. An initial cranial MR scan did not indicate any anomalies (Figure 1A). Further, the head and neck CTA revealed a stenosed right middle cerebral artery and a barely discernible constriction at the ostium of the left vertebral artery. Lumbar puncture indicated a pressure of 230 mmH_2_O. The cerebrospinal fluid had leukocytes at 190 × 10^6^/L (reference range: 0–8 × 10^6^/L) and protein levels at 0.54 g/L (reference range: 0.2–0.4 g/L). CSF mNGS identified 4 sequences of Epstein–Barr virus (EBV). Viral cerebellar encephalitis was suspected, but despite antiviral therapy, there was no symptomatic improvement, prompting his visit to our hospital.

Upon examination, his vitals were recorded as: temperature 36.6°C, pulse rate 70 bpm, respiratory rate 19 breaths per minute, and blood pressure at 141/92 mmHg. Cardio-respiratory and abdominal assessments were unremarkable. Neurological evaluation indicated clear consciousness, coherent speech, horizontal nystagmus in both eyes, imprecise bilateral finger-nose and heel-shin tests, positive Romberg sign, and no other evident abnormalities. A preliminary diagnosis suggested cerebellar encephalitis, and a treatment regimen of Acyclovir combined with Dexamethasone (10 mg) was initiated.

Post-admission, standard blood tests, biochemistry, coagulation profile, D-dimer, myocardial enzymes, BNP, thyroid function and antibodies, PCT, ESR, CRP, tumor markers, and TORCH were all found to be within normal limits. A repeat lumbar puncture yielded a pressure of 210 mmH_2_O, white blood cells at 50 × 10^6^/L, protein at 0.50 g/L, with cerebrospinal fluid cytology predominantly indicating a lymphocytic response. Both the cerebrospinal fluid and serum tested negative for a series of autoimmune encephalitis antibodies (anti-NMDAR, AMPAR1, AMPAR2, LGI1, CASPR2, GABABR, GAD65), paraneoplastic neurological syndrome antibodies (Hu, Yo, Ri, Amphiphysin、Ma2, CV2/CRMP5), and ganglioside antibodies (GM1-IgG, GD1b-IgG, GQ1b-IgG, GM1-IgM, GD1b-IgM, GQ1b-IgM).

**Figure 2:**
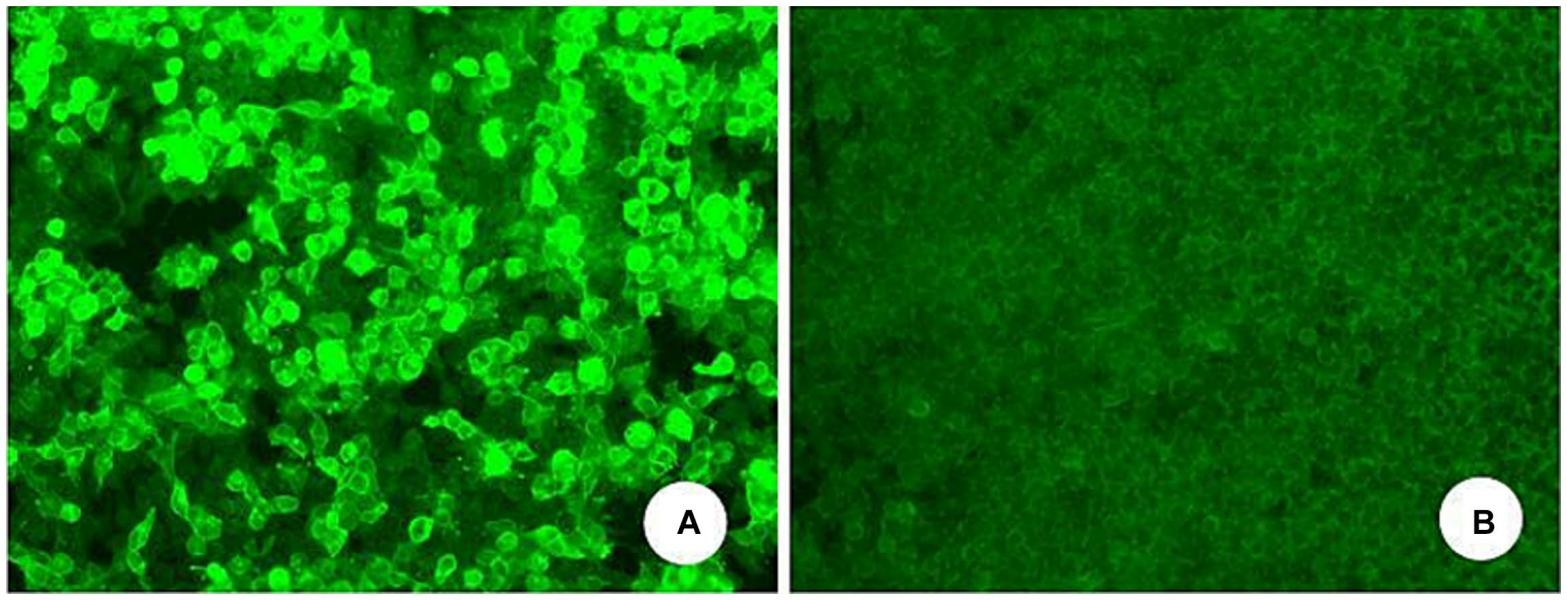
Serum anti-mGluR1-IgG positive **(A)** and negative control **(B)** of another serum individuals sample (transfected cell method).

During hospitalization, the patient’s condition deteriorated, exhibiting sleep disturbances and altered mental behavior. A treatment regimen comprising Olanzapine, Eszopiclone, intravenous human immunoglobulin (0.4 g/kg for 5 days), and methylprednisolone sodium succinate (500 mg for 3 days) was administered. The patient was discharged after showing improvement. However, on 1 May 2020, he experienced exacerbation of dizziness and unsteady walking, with a new symptom of coughing when drinking water. A subsequent head MRI did not reveal any discernible abnormalities. Both serum autoimmune encephalitis antibody and serum AQP-4 tests were negative. Further examination of cerebellar encephalitis antibody profile at Peking Union Medical College Hospital revealed the presence of serum anti-mGluR1 with an end-point titer of 1:1,000 (Figure 2), leading to a definitive diagnosis of Anti-mGluR1 encephalitis. The patient was readmitted to our facility, receiving Human Immunoglobulin (0.4 g/kg for 5 days), Methylprednisolone (500 mg for 3 days followed by a tapering regimen), Mycophenolate Mofetil (0.5 g twice daily), and Olanzapine (5 mg nightly). Although the patient’s psychiatric symptoms improved, there was negligible enhancement in cerebellar ataxia. Post-discharge, he continued rehabilitation exercises with periodic follow-ups. A subsequent review on 14 April 2023, revealed an mRS score of 3, and a cranial MRI indicated cerebellar atrophy (Figure 1B).

## Discussion

In the presented case, the patient’s initial symptoms, characterized by clumsiness in speech and unstable gait, were accompanied by a prodromal infection. Physical examination confirmed the presence of cerebellar syndrome. The progression of the illness saw the patient manifesting psycho-behavioral disturbances and cognitive impairments. An initial MRI scan did not reveal notable abnormalities. Lumbar puncture indicated elevated cerebrospinal fluid pressure, increased white blood cell count, and elevated protein levels. The next-generation sequencing of cerebrospinal fluid revealed a viral infection, leading to a presumptive diagnosis of viral cerebellar encephalitis. However, given the ineffectiveness of the antiviral therapy, the differential diagnosis was refined to consider immune-related cerebellar encephalitis. This led to an extended immune-related antibody profile test, confirming the presence of anti-mGluR1 with an end-point titer of 1:1,000, and culminating in a definitive diagnosis of anti-mGluR1 encephalitis.

Smitt et al. (3) first documented two anti-mGluR1 encephalitis cases, where both individuals exhibited cerebellar ataxia and had a prior history of Hodgkin lymphoma. To date, literature has recorded 36 such cases globally. Research by Khojah et al. (4) highlighted that 25% of patients experienced one or more prodromal symptoms—ranging from fever, headache, and fatigue to weight loss, nausea, vomiting, night sweats, and flu-like manifestations—with a median interval of 30 days leading up to neurological symptom onset. In a literature review, three patients reported prior infections, including a trigeminal herpes zoster infection a month before onset (5), a streptococcal pharyngitis 2 months prior (6), and a dengue virus infection (7). The link between such infections and anti-mGluR1 encephalitis remains ambiguous. These findings hint at potential post-infectious factors contributing to the development of anti-mGluR1 encephalitis, or the possibility that infections could catalyze its pathogenesis (4). EBV was detected in the cerebrospinal fluid of patients. As the first discovered human oncovirus, EBV infects more than 90% of the world’s people, and once infected, most will remain latent in B lymphocytes in an asymptomatic form of infection and cannot be cleared (8). Infection leads to a spectrum of EBV-associated diseases when the balance between the virus and the host is disrupted (9). The relationship between viral infection and cerebellar encephalitis in this patient needs further investigation.

Over the recent years, it’s been reported that 27% of patients with herpes simplex virus encephalitis (HSE) subsequently develop secondary autoimmune encephalitis (AE), which includes predominant cases of anti-NMDAR encephalitis (64%) and other variations such as GABAbR encephalitis, LGI1 encephalitis, and AMPAR encephalitis (10). Notably, 30% of patients without neurological symptoms post-HSE infection demonstrated autoantibodies, including NMDAR (27%) and other antibodies (73%) in serum and/or cerebrospinal fluid. This case study postulates that the emergence of anti-mGluR1 encephalitis in the presented patient might be associated with preceding viral infection. Several hypotheses abound regarding the etiology of autoimmune encephalitis post-HSV (herpes simplex virus) infection (11): (1) Molecular mimicry, where exogenous pathogen antigens structurally resemble the host’s own antigens, resulting in specific antibodies or effector T cells produced against these exogenous antigens inadvertently cross-reacting with the host’s analogous antigens, leading to autoimmune repercussions. (2) The release of autoantigens from disintegrated neurons post-viral infection, which disrupts central immune tolerance (12). (3) Autoinflammatory responses against the herpes virus infection, where HSV triggers T and B cell activation, producing a cascade of inflammatory cytokines. These might infiltrate the blood-cerebrospinal fluid barrier, instigating immune responses in the CNS and subsequently recognizing CNS autoantigens (13). (4) Genetic predispositions (14). (5) Secondary immunodeficiencies. For instance, HIV infections might induce an immune response dysregulation to NMDAR, culminating in the emergence of AEs (15). However, the nexus between anti-mGluR1 encephalitis, its prodromal infections, and its specific mechanisms remains nebulous and warrants further in-depth research.

In a retrospective analysis encompassing 36 cases of anti-mGluR1 encephalitis (4), 16.7% of patients had diagnoses of unrelated autoimmune diseases to mGluR1 antibodies, including conditions like multiple sclerosis, Hashimoto’s thyroiditis, Sjögren’s syndrome, and pernicious anemia. Furthermore, 22.2% had associated malignancies, with six cases being lymphomas. Notably, 50% of these malignancy cases manifested autoimmune cerebellar or encephalitis within 5 years of onset. Iorio et al. (16) reported a unique case of anti-mGluR1 encephalitis associated with prostate cancer. Immunohistochemistry of the patient’s prostate adenocarcinoma revealed significant mGluR1 expression in the tumor’s luminal acinar epithelial cells. Additionally, patient IgG was observed binding to tumor mGluR1. Intriguingly, mGluR1 receptor expression has been substantiated in prostate cancer, with its expression correlating with the progression of the malignancy (17). Additionally, mGluR1 expression is evident in human cutaneous T-cell lymphoma cell lines (18). While it’s uncertain if malignancies play a direct role in instigating anti-mGluR1 encephalitis, it’s paramount for physicians to undertake tumor-related assessments, including tumor markers and PET-CT scans in such patients. As of now, this patient has not demonstrated autoimmune diseases unrelated to mGluR1 antibodies or malignant tumors. Continuous observation and regular follow-ups are crucial.

The clinical presentation of this patient centered on cerebellar syndrome. In the literature, anti-mGluR1 encephalitis manifests a spectrum of symptoms, including gait instability, cerebellar dysarthria, abnormal eye movements, and limb ataxia (Table 1). As the disease unfolds, the majority of patients exhibit behavioral changes, cognitive impairments, taste dysfunction, autonomic dysregulation, seizures, and sleep disorders. Rarer clinical manifestations encompass visual disturbances and limb weakness (19). Dyskinesias, when manifested, present as myoclonus or dystonia in adults, while children predominantly exhibit athetosis (20).

**Table 1:** Basic information, clinical symptoms and laboratory tests of 36 patients diagnosed with anti-mGluR1 encephalitis in the literature.

Demographic features	Number of patients
Median age	52.5 (3,81)
**Gender**	
Male	19
Female	17
**Clinical manifestations**	
Cerebellar symptoms	**34**
Ataxia	31
Dysarthria	19
Nystagmus	10
Titubation	7
Dysmetria	7
Vertigo	6
Diplopia	4
Intention tremor	4
Oscillopsia	2
Behavioral symptoms	10
Cognitive symptoms	10
Sleep difficulties	10
**CSF features**	
Leukocytes/μL	30
Normal (0–5)	15
Pleocytosis (>5)	15
Median	5.5 (0,214)
Oligoclonal Bands	22
Negative	10
Positive	12
Brain MRI	**33**
Normal	9
Cerebellar atrophy	15
Cerebellar hyperintensity	7
Spinal cord lesions	2
Other lesions	5

CSF, cerebrospinal fluid; MRI, magnetic resonance imaging.

For the anti-mGluR1, detection rates in serum and cerebrospinal fluid stood at 97% and 95%, respectively (19). In some cases, the antibody was detected solely either in serum or cerebrospinal fluid. For our particular patient, only the serum was tested, as he opted against a repeated lumbar puncture. Notably, NMDA-R, LGI1 and CASPR2 encaphalitis, typically present with CSF cell numbers in the range of 0–20 leukocytes/μL. Our patient had 190 leukocytes/μL in CFS, which was significantly higher than other typical autoimmune encephalitis. We reviewed the literature and found that the number of CSF cells in patients with mGLuR1 encephalitis, which has been reported so far, ranges from 0 to 214 leukocytes/μL. And nearly half of such patients demonstrated leukocytosis in the cerebrospinal fluid, accompanied by specific oligoclonal bands or an increased IgG index.

Electroencephalography (EEG) can reveal bilateral focal frontotemporal slow waves, potentially associated with epileptiform discharges (6). Though early imaging displayed abnormalities in merely one-third of the patients, encompassing findings like brain atrophy, variable brain and spinal cord lesions, and cerebellar abnormalities such as cerebellar hyperintensity, leptomeningeal enhancement, atrophy, or edema, these typically were concentrated in the medial cerebellar hemisphere and vermis (4). Intriguingly, as time progressed, positive MRI results were observed in three-quarters of these patients. Such deviations in MRI results, transitioning from normal to abnormal, are attributed to the degeneration of Purkinje cells due to prolonged antibody exposure (21)—a shift highlighting the imperative of timely intervention. PET scans are instrumental in excluding hidden malignancies. In line with the guidelines from the European Federation of Neurological Societies, a PET-CT follow-up is strongly advised in instances with heightened suspicion of a paraneoplastic syndrome (22). In the context of our patient, the initial cranial MRI did not present any abnormalities. Despite this, post-initiation of the immunomodulatory therapy, while the patient’s clinical symptoms remained stable over the span of 3 years, subsequent MRI scans indicated cerebellar atrophy. This evokes concerns about the long-term prognosis for patients with anti-mGluR1 encephalitis.

For most individuals diagnosed with anti-mGluR1 antibody-associated encephalitis, first-line immunotherapy treatments such as steroids, plasma exchange, and intravenous immunoglobulins are prescribed. According to a study by Khojah et al. (4), 41.7% of these patients progressed to second-line therapy, including agents such as rituximab, azathioprine, cyclophosphamide, mycophenolate mofetil, tacrolimus, and hydroxychloroquine. Among these, 93.3% did not achieve full remission with the second-line treatment, and 36.1% utilized more than three distinct treatment modalities. Even then, merely 15.4% managed to attain complete remission. Comparatively, patients with anti-mGluR1 encephalitis tend to have a grimmer prognosis than those with other autoimmune encephalitides, such as anti-mGluR5 encephalitis, anti-NMDAR encephalitis, or anti-LGI1 encephalitis (6, 23, 24). Independent of the underlying cause of anti-mGluR1 encephalitis, commencing treatment at an early stage remains pivotal.

## Conclusion

In summation, when encountering patients with acute or subacute onset prominently manifesting cerebellar syndromes—and where common autoimmune and paraneoplastic antibody profiles return negative—screening for the rarer anti-mGluR1 becomes imperative. A minority of these patients might harbor tumors. Acute-phase cerebrospinal fluid evaluations might display mildly elevated leukocyte counts. A definitive diagnosis necessitates the detection of anti-mGluR1-IgG in serum and/or cerebrospinal fluid. It’s evident that this disease exhibits a limited response to immunotherapy, underscoring the importance of early therapeutic interventions. Upcoming research endeavors should aim to elucidate the intricate relationship between anti-mGluR1 antibody-associated encephalitis, prodromal infections, and tumors. Additionally, we must embark on the quest to uncover more effective treatment regimens.

## Data availability statement

The original contributions presented in the study are included in the article/supplementary material, further inquiries can be directed to the corresponding authors.

## Ethics statement

Ethical review and approval was not required for the study on human participants in accordance with the local legislation and institutional requirements. Written informed consent from the patients/participants or patients/participants' legal guardian/next of kin was not required to participate in this study in accordance with the national legislation and the institutional requirements. Written informed consent was obtained from the individual(s) for the publication of any potentially identifiable images or data included in this article.

## Author contributions

XC: Conceptualization, Data curation, Investigation, Writing – original draft. YaC: Conceptualization, Funding acquisition, Investigation, Methodology, Writing – original draft. LD: Investigation, Methodology, Writing – original draft. NL: Methodology, Project administration, Writing – review & editing. TL: Formal analysis, Methodology, Writing – review & editing. YuC: Resources, Supervision, Validation, Writing – review & editing. WD: Funding acquisition, Methodology, Supervision, Validation, Writing – review & editing.

## References

[1] Scotton WJ Karim A Jacob S. "Glutamate receptor antibodies in autoimmune central nervous system disease: Basic mechanisms, clinical features, and antibody detection,". New York, NY: Springer New York (2019). p. 225–255. 10.1007/978-1-4939-9077-1_15 30707437

[2] Benarroch EE. Metabotropic glutamate receptors: synaptic modulators and therapeutic targets for neurologic disease. Neurology. (2008) 70:964–8. doi: 10.1212/01.wnl.0000306315.03021.2a 18347319

[3] Smitt PS Kinoshita A De Leeuw B Moll W Coesmans M Jaarsma D. Paraneoplastic cerebellar Ataxia due to autoantibodies against a glutamate receptor. N Engl J Med. (2000) 342:21–7. doi: 10.1056/NEJM200001063420104 10620645

[4] Khojah O Makkawi S Alghamdi S. Anti-mGluR1 encephalitis: case illustration and systematic review. Front Neurol. (2023) 14:1142160. doi: 10.3389/fneur.2023.1142160, PMID: 37139064 PMC10149714

[5] Lopez-Chiriboga AS Komorowski L Kumpfel T Probst C Hinson SR Pittock SJ. Metabotropic glutamate receptor type 1 autoimmunity: clinical features and treatment outcomes. Neurology. (2016) 86:1009–13. doi: 10.1212/WNL.0000000000002476, PMID: 26888994 PMC4799712

[6] Spatola M Petit Peol M Maudes E Simabukuro M Muñiz-Castrillo S Pinto AL. Clinical features, prognostic factors, and antibody effects in anti-mGluR1 encephalitis. Neurology. (2020) 95:e3012–25. doi: 10.1212/WNL.0000000000010854, PMID: 32928978 PMC7734921

[7] Chaumont H Petit A Mameri T Schollhammer R Honnorat J Lannuzel A. Successful management of anti-mGluR1 encephalitis with immunosuppressive treatment: Dengue virus as a trigger? Move Disord Clin Pract. (2019) 6:727–8. doi: 10.1002/mdc3.12841 PMC6856456 31745489

[8] Nowalk A Green M. Epstein-Barr Virus. Microbiol Spectr. (2016) 4:2015. doi: 10.1128/microbiolspec.DMIH2-0011-2015 27337443

[9] Taylor GS Long HM Brooks JM Rickinson AB Hislop AD. The immunology of Epstein-Barr virus-induced disease. Annu Rev Immunol. (2015) 33:787–821. doi: 10.1146/annurev-immunol-032414-112326 25706097

[10] Armangue T Spatola M Vlagea A Mattozzi S Cárceles-Cordon M Martinez-Heras E. Frequency, symptoms, risk factors, and outcomes of autoimmune encephalitis after herpes simplex encephalitis: a prospective observational study and retrospective analysis. Lancet Neurol. (2018) 17:760–72. doi: 10.1016/S1474-4422(18)30244-8, PMID: 30049614 PMC6128696

[11] Gelfand JM. Autoimmune encephalitis after herpes simplex encephalitis: insights into pathogenesis. Lancet Neurol. (2018) 17:733–5. doi: 10.1016/S1474-4422(18)30279-5 30049613

[12] Alexopoulos H Akrivou S Mastroyanni S Antonopoulou M Dinopoulos A Giorgi M. Postherpes simplex encephalitis: a case series of viral-triggered autoimmunity, synaptic autoantibodies and response to therapy. Ther Adv Neurol Disord. (2018) 11:175628641876877. doi: 10.1177/1756286418768778, PMID: 29774053 PMC5949951

[13] Liu J Liu L Kang W Peng G Yu D Ma Q. Cytokines/chemokines: potential biomarkers for non-paraneoplastic anti-N-methyl-D-aspartate receptor encephalitis. Front Neurol. (2020) 11:582296. doi: 10.3389/fneur.2020.582296, PMID: 33408682 PMC7779630

[14] Gnann JW Jr Whitley RJ. Herpes simplex encephalitis an update. Curr Infect Dis Rep. (2017) 19:13. doi: 10.1007/s11908-017-0568-7, PMID: 28251511

[15] Cunill V Arboleya S Jiménez MDLR Campins A Herbera P Mestre L. Neuronal surface antibodies in HIV-infected patients with isolated psychosis. J Neuroimmunol. (2016) 301:49–52. doi: 10.1016/j.jneuroim.2016.10.008, PMID: 27836183

[16] Iorio R Damato V Mirabella M Vita MG Hulsenboom E Plantone D. Cerebellar degeneration associated with mGluR1 autoantibodies as a paraneoplastic manifestation of prostate adenocarcinoma. J Neuroimmunol. (2013) 263:155–8. doi: 10.1016/j.jneuroim.2013.07.015 23958353

[17] Koochekpour S Majumdar S Shourideh M Rezaei K Sartor O Mohler JL. Serum glutamate levels correlate with Gleason score and glutamate blockade decreases proliferation, migration, and invasion and induces apoptosis in prostate Cancer cells. Clin Cancer Res. (2012) 18:5888–901. doi: 10.1158/1078-0432.CCR-12-1308, PMID: 23072969 PMC3492499

[18] Chiocchetti A Miglio G Mesturini R Varsaldi F Mocellin M Orilieri E. Group I mGlu receptor stimulation inhibits activation-induced cell death of human T lymphocytes. Br J Pharmacol. (2006) 148:760–8. doi: 10.1038/sj.bjp.0706746, PMID: 16751798 PMC1617076

[19] Lei L Jianguo L Zhang Jingxiao Y Yingxin QX Jiawei W. A case of antimetabotropic glutamate receptor 1 antibody-associated encephalitis. Chin J Neurol. (2021) 54:104. doi: 10.3760/cma.j.cn113694-20210208-00104

[20] Chandler E Arvantis N Morgan B. A novel case of idiopathic MGluR1 encephalitis in a pediatric patient. Child Neurol Open. (2022) 9:2329048X2210956–221095695X. doi: 10.1177/2329048X221095695, PMID: 35497371 PMC9047037

[21] Yoshikura N Kimura A Fukata M Fukata Y Yokoi N Harada N. Long-term clinical follow-up of a patient with non-paraneoplastic cerebellar ataxia associated with anti-mGluR1 autoantibodies. J Neuroimmunol. (2018) 319:63–7. doi: 10.1016/j.jneuroim.2018.04.001 29685291

[22] Bresler R Schroeder HWI Chow DZ Lim R. 18F-fluorodeoxyglucose positron emission tomography/computed tomography in the diagnosis of suspected paraneoplastic syndromes: a retrospective analysis. World J Nucl Med. (2020) 19:124–30. doi: 10.4103/wjnm.WJNM_48_19 32939199 PMC7478297

[23] van Sonderen A Thijs RD Coenders EC Jiskoot LC Sanchez E de Bruijn MA. Anti-LGI1 encephalitis: clinical syndrome and long-term follow-up. Neurology. (2016) 87:1449–56. doi: 10.1212/WNL.0000000000003173 27590293

[24] Spatola M Sabater L Planagumà J Martínez-Hernandez E Armangué T Prüss H. Encephalitis with mGluR5 antibodies: symptoms and antibody effects. Neurology. (2018) 90:e1964–72. doi: 10.1212/WNL.0000000000005614 29703767 PMC5980520

